# Diagnostic performance and clinical applications of artificial intelligence for intracranial bleeding detection: A meta-analysis

**DOI:** 10.1016/j.bas.2025.105866

**Published:** 2025-11-10

**Authors:** Mustafa S. Alhasan, Ahmed Y. Azzam, Ayman S. Alhasan, Arjun Kalyanpur, Omar A. Alharthi, Mohammad Khalil, Adam Dmytriw, Muhammed Amir Essibayi, Fabricio Feltrin, James Milburn

**Affiliations:** a Department of Internal Medicine, College of Medicine, Taibah University, Madinah, Saudi Arabia; b Consultant Teleradiologist, Teleradiology Solutions, 22 Llanfair Rd UNIT 6, Ardmore, PA, 19003, USA; c Department of Neuroradiology, Rockefeller Neuroscience Institute, West Virginia University, Morgantown, WV, USA; d Department of Radiology, Teleradiology Solutions, 22 Llanfair Rd UNIT 6, Ardmore, PA, 19003, USA; e Faculty of Medicine, King Abdulaziz University, Jeddah, Saudi Arabia; f Neurovascular Centre, Departments of Medical Imaging & Neurosurgery, St. Michael's Hospital, University of Toronto, Toronto, Ontario, Canada; g Neuroendovascular Program, Massachusetts General Hospital & Brigham and Women's Hospital, Harvard Medical School, Boston, MA, USA; h Montefiore-Einstein Cerebrovascular Research Lab, Montefiore Medical Center, Albert Einstein College of Medicine, Bronx, NY, USA; i Department of Neurological Surgery, Montefiore Medical Center, Albert Einstein College of Medicine, Bronx, NY, USA; j Division of Radiology - Neuroradiology, UT Southwestern Medical Center, University of Texas Southwestern, 5323 Harry Hines Blvd, Dallas, TX, 75390, USA; k The University of Queensland Medical School, Ochsner Clinical School, New Orleans, LA, USA; l Department of Radiology, Ochsner Clinic Foundation, New Orleans, LA, USA

**Keywords:** Artificial intelligence, Deep learning, Intracranial hemorrhage, Computed tomography, Diagnostic accuracy, Neuroimaging

## Abstract

**Introduction:**

Intracranial hemorrhage (ICH) is a neurological emergency with high mortality rates requiring timely diagnosis. While computed tomography (CT) remains the gold standard, diagnostic accuracy varies with radiologist experience and workload. This systematic review and meta-analysis aims to evaluate the diagnostic performance of AI algorithms in detecting ICH on CT imaging and to explore key considerations for their clinical implementation in emergency and teleradiology settings.

**Methods:**

We conducted a systematic review and meta-analysis following PRISMA-DTA guidelines, searching seven databases up to May 2025. Studies evaluating AI diagnostic accuracy for ICH detection on non-contrast CT scans were included. Quality assessment used QUADAS-2 criteria. Pooled estimates were calculated using random-effects models, with subgroup analyses by algorithm architecture and ICH subtype.

**Results:**

A total of 45 studies met the inclusion criteria, comprising 29 research algorithm evaluations (n = 185,847 patients) and 16 studies of commercial AI system implementations (n = 94,523 patients). Research algorithms demonstrated a pooled sensitivity of 0.890 (95 % CI: 0.839–0.942) and specificity of 0.926 (95 % CI: 0.899–0.954). Commercial AI systems exhibited slightly superior performance, with sensitivity of 0.899 (95 % CI: 0.858–0.940) and specificity of 0.951 (95 % CI: 0.928–0.974). Diagnostic accuracy varied notably across ICH subtypes, with epidural hemorrhage presenting the greatest detection challenge (difficulty score: 0.251). Among algorithmic designs, convolutional recurrent neural networks (CNN-RNNs) demonstrated the highest diagnostic performance. In real-world clinical implementation, AI integration demonstrated substantial workflow improvements: door-to-treatment decision time reduced by 26 % (92 → 68 min), critical case notification time decreased by 57 % (75 → 32 min), and triage accuracy improved by 8 % (86 %→94 %), directly impacting patient care pathways. Despite a 7–8 % sensitivity reduction compared to benchmark settings, these clinical benefits were consistent across implementations.

**Conclusions:**

AI algorithms demonstrate strong diagnostic performance in detecting ICH, with commercial systems demonstrating superior specificity compared to research models. Despite notable performance gaps in detecting certain hemorrhage subtypes, particularly epidural hemorrhage, the clinical benefits of AI integration, including improved workflow efficiency and reduced time to treatment decisions, are substantial. Future research should prioritize prospective validation and the development of algorithms tailored to enhance detection across challenging ICH subtypes.

## Highlights

• AI algorithms achieve 89–90 % sensitivity and 93–95 % specificity for detecting brain bleeding on CT scans, matching or exceeding human radiologist performance.
• AI struggles most with epidural hemorrhage detection (75 % sensitivity) but excels at detecting intraparenchymal bleeding (95 % sensitivity).
• AI implementation reduces door-to-treatment decision time by 26 % and critical case notification time by 57 % in real-world clinical settings.

## 1 Introduction

Intracranial hemorrhage (ICH) is a neurological emergency associated with high morbidity and mortality, occurring in approximately 25 cases per 100,000 persons annually and accounting for nearly two million stroke cases worldwide (Wang et al., 2022; Hurford et al., 2020). Timely and accurate diagnosis is important as the prognosis of outcomes is significantly linked and improved with early intervention, especially within the first hours after onset (Mun and Hinman, 2022). Computed tomography (CT) is considered to be the first-line gold standard imaging modality for ICH detection due to its availability, rapid acquisition time, and high sensitivity for acute bleeding detection (Romero and Rojas-Serrano, 2023). However, the interpretation of head CT scans requires specialized expertise, and diagnostic accuracy can vary with each radiologist experience, workload, and fatigue. These challenges are further burdened by increasing imaging volumes and workforce shortages in many healthcare systems (Yeo et al., 2023).

Artificial intelligence (AI) modalities, including both machine learning and deep learning algorithms, have emerged as promising tools to augment radiological practice in the detection of intracranial hemorrhage (Kundisch et al., 2021). AI-powered systems can assist in analyzing imaging data, identifying hemorrhagic patterns, reducing interpretation time, and potentially improving diagnostic accuracy. In recent years, there has been a proliferation of studies evaluating various AI algorithms for ICH detection, with reported sensitivities and specificities often exceeding 90 %. However, substantial variability exists in algorithmic architectures, validation methodologies, and performance metrics across different hemorrhage subtypes (Babi et al., 2025).

Beyond diagnostic accuracy, the clinical value of AI systems depends critically on their impact on time-sensitive workflows. In neurosurgical emergencies, delays in ICH detection directly correlate with adverse patient outcomes, with each hour of delay associated with increased mortality and disability. Key clinical implementation questions include: How do AI systems affect door-to-treatment decision times? What is their role in emergency department triage? How do they integrate with existing radiology workflows? How do predictive values vary across different clinical settings and patient populations? This meta-analysis aims to investigate these questions alongside traditional diagnostic accuracy metrics.

Despite the evidence from prior studies and the expanding body of literature, several key knowledge gaps remain that hinder more targeted and effective clinical implementation (Babi et al., 2025). First, the comparative performance of different algorithmic architectures across various ICH subtypes remains inadequately clarified. Second, the translation gap between benchmark dataset performance and real-world clinical effectiveness has not been thoroughly quantified. Third, the clinical implications of algorithm performance for specific applications remain poorly documented. Additionally, the temporal evolution of AI capabilities in the context of ICH detection has yet to be comprehensively characterized (Ai et al., 2024).

To address these gaps, this systematic review and meta-analysis aimed to answer four specific research questions. First, what is the overall diagnostic performance of AI algorithms for ICH detection, and how do research algorithms compare to commercial systems? Second, how does diagnostic accuracy vary across ICH subtypes, and which hemorrhage types pose the greatest detection challenges? Third, what is the performance gap between benchmark dataset evaluations and real-world clinical implementation? Fourth, what is the quantifiable impact of AI implementation on clinical workflow metrics, including door-to-treatment decision and triage accuracy? By addressing these questions, we provide evidence-based guidance for clinical implementation and identify priorities for future algorithm development.

## 2 Methods

### 2.1 Study design and search strategy

We conducted our study in accordance with the Preferred Reporting Items for Systematic Reviews and Meta-Analyses for Diagnostic Test Accuracy Studies (PRISMA-DTA) guidelines (McInnes et al., 2018). A comprehensive literature search was performed across seven databases, PubMed/MEDLINE, EMBASE, Web of Science, Scopus, Cochrane Library, CENTRAL, and Google Scholar covering publications up to May 29, 2025. The search strategy included a combination of Medical Subject Headings (MeSH) and free-text keywords related to artificial intelligence, machine learning, deep learning, intracranial hemorrhage, and diagnostic accuracy. In addition, we manually screened the reference lists of included studies and relevant reviews to identify further eligible articles.

Search terms were customized to capture records involving artificial intelligence, intracranial hemorrhage, and diagnostic performance. For artificial intelligence, the search included terms such as: artificial intelligence, machine learning, deep learning, neural network, convolutional neural network (CNN), deep neural network (DNN), computer vision, computer-assisted, automated detection, algorithm, computer-aided, AI, ML, DL, transfer learning, and supervised learning. For intracranial hemorrhage, terms included: intracranial hemorrhage, brain hemorrhage, cerebral hemorrhage, ICH, intraparenchymal hemorrhage (IPH), subarachnoid hemorrhage (SAH), subdural hemorrhage (SDH), epidural hemorrhage (EDH), intraventricular hemorrhage (IVH), intracerebral hemorrhage, cerebral bleeding, and brain bleeding. For diagnostic performance, search terms included: diagnosis, detect, identify, recognize, characterize, classify, classification, accuracy, sensitivity, specificity, receiver operating characteristic (ROC), area under the curve (AUC), precision, recall, F1 score, diagnostic performance, and CT scan.

### 2.2 Eligibility criteria and study selection

We included studies that evaluated the diagnostic accuracy of AI algorithms for detecting ICH on non-contrast CT scans, using radiologist reports or consensus readings as the reference standard. Studies were considered eligible if they reported sufficient data to calculate sensitivity and specificity, or if these metrics were provided directly in an extractable format. We excluded studies that focused exclusively on magnetic resonance imaging (MRI), contrast-enhanced CT, or that evaluated only post-treatment hemorrhage or hemorrhage quantification without detection. Conference abstracts were also excluded.

### 2.3 Data extraction and quality assessment

The extracted data from eligible studies included publication details (authors, year, country), study characteristics (design, sample size, ICH subtypes evaluated), AI algorithm specifications (architecture type, training methodology), validation approach (internal or external), and diagnostic performance metrics (sensitivity, specificity, AUC, and accuracy). For studies reporting algorithm performance by ICH subtype or comparing multiple models, we also extracted subtype-specific and algorithm-specific performance metrics. The methodological quality of included studies was assessed using the Quality Assessment of Diagnostic Accuracy Studies-2 (QUADAS-2) tool, which evaluates the risk of bias across four domains: patient selection, index test, reference standard, and flow and timing.

### 2.4 Data synthesis and statistical analysis

We calculated pooled estimates of sensitivity, specificity, and AUC using a random-effects model to account for inter-study heterogeneity. For studies that reported results from multiple algorithms or across different ICH subtypes, we performed separate meta-analyses stratified by algorithm type and hemorrhage subtype. Ninety-five percent confidence intervals (CIs) were calculated for all pooled estimates. Heterogeneity was assessed using the I^2^ statistic, with thresholds of 25 %, 50 %, and 75 % indicating low, moderate, and high heterogeneity, respectively. Publication bias was evaluated through visual inspection of funnel plot asymmetry.

We performed several subgroup analyses to explore sources of heterogeneity and address key research objectives (Wang et al., 2022): comparison of algorithm architectures (deep learning versus traditional machine learning) (Hurford et al., 2020); focus on specific ICH subtypes (Mun and Hinman, 2022); benchmark dataset performance versus real-world clinical performance (Romero and Rojas-Serrano, 2023); data source comparison (single-center versus multi-center studies); and (Yeo et al., 2023) temporal trends based on publication year. For ICH subtypes, we calculated a “detection difficulty score” (1 − sensitivity) to quantify the relative difficulty of detecting each hemorrhage subtype. For algorithm–subtype interactions, we developed a performance matrix to evaluate diagnostic metrics across different combinations and identify optimal algorithms for specific subtypes. Meta-regression was conducted to assess the influence of study-level covariates on diagnostic accuracy. All statistical analyses were performed using RStudio with R version 4.4.2 (R Foundation for Statistical Computing, Vienna, Austria) and the “mada,” “metafor,” and “meta” packages.

## 3 Results

### 3.1 Study selection and characteristics

**Fig. 1:**
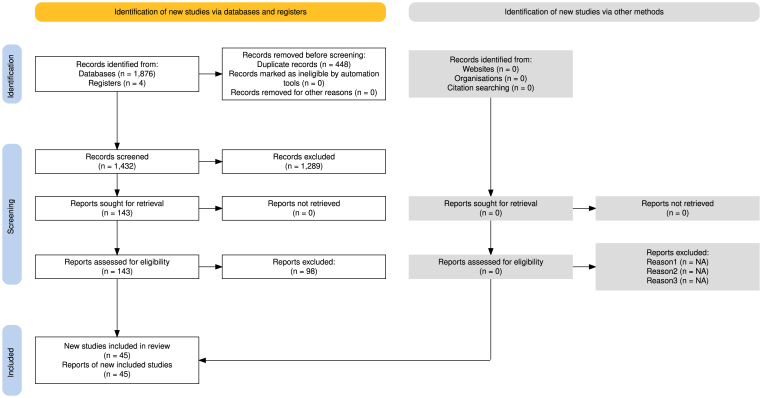
PRISMA flowchart of included studies process.

Our literature search, conducted from inception to May 29, 2025, identified a total of 45 studies that met the inclusion criteria for this systematic review and meta-analysis (Fig. 1). These comprised 29 studies focused on research algorithm development and validation, and 16 studies evaluating the implementation of commercial AI systems. The included studies originated from diverse geographic regions, including North America, Europe, and the Asia-Pacific (Table 1).

**Table 1:** Baseline characteristics and demographics of included studies.

Author, Year	Country	Study Design	Sample Size	Algorithm Type	ICH Subtypes	Data Source	Sensitivity/Specificity	AUC	Validation Method
**Research Algorithm Development and Validation Studies:**									
Schmitt et al., 2022 (Schmitt et al., 2022)	Germany	Retrospective	78	CNN	ICH	Single center	0.91/0.89	0.90	Internal
Phaphuangwittayakul et al., 2022 (Phaphuangwittayakul et al., 2022)	China	Retrospective	458	CNN	ICH, EDH, SDH, IPH	Single center	0.96/0.97	–	Internal
Hopkins et al., 2022 (Hopkins et al., 2022)	USA	Prospective	112,695	DNN	ICH	Single center	0.98/0.99	0.99	External
Seyam et al., 2022 (Seyam et al., 2022b)	Switzerland	Prospective	431	DL	ICH	Single center	0.87/0.94	–	Internal
Altuve and Pérez, 2022 (Altuve and Pérez, 2022)	Venezuela	Retrospective	100	ResNet-18	ICH	Single center	0.96/0.96	–	Internal
Tang et al., 2022 (Tang et al., 2022)	China	Retrospective	5	CNN	ICH	Single center	0.92/0.88	–	Internal
Cortes-Ferre et al., 2022 (Cortés-Ferre et al., 2023b)	Spain	Retrospective	3497	DL	ICH	Single center	0.91/0.94	0.98	Internal
Kau et al., 2022 (Kau et al., 2022)	Austria	Retrospective	2139	DL	ICH	Single center	0.68/0.97	–	Internal
Tharek et al., 2022 (Tharek et al., 2022)	Malaysia	Retrospective	102	CNN	ICH	Single center	0.97/0.93	–	Internal
Abe et al., 2022 (Abe et al., 2022)	Japan	Retrospective	259	XGBoost	ICH	Single center	0.74/0.75	0.80	Internal
Trevisi et al., 2022 (Trevisi et al., 2022)	Italy	Retrospective	259	RF	ICH	Multiple centers	0.78/0.86	0.93	Internal
Uchida et al., 2022 (Uchida et al., 2022)	Japan	Prospective	2734	LR, RF, XGBoost	ICH, SAH	Multiple centers	0.43/0.92∗	0.82∗	External
Alis et al., 2022 (Alis et al., 2022)	Turkey	Retrospective	121,436	CNN-RNN	ICH, IPH, IVH, SAH, SDH, EDH	Multiple centers	0.96/0.96	0.96	Internal
Rao et al., 2022 (Rao et al., 2022)	India	Retrospective	2288	Multiple∗∗	ICH	Single center	0.99/1.00∗∗∗	1.00∗∗∗	Internal
Zhou et al., 2022 (Zhou et al., 2022)	China	Retrospective	5088	ResNet-18, DenseNet-121	EDH, IVH, CPH, SAH, SDH	Single center	0.98/0.88†	–	Internal
Salehinejad et al., 2021 (Salehinejad et al., 2021)	Canada	Retrospective	2428	SE-ResNeXt	EDH, SDH, SAH, IVH, IPH	Single center	††	††	External
McLouth et al., 2021 (McLouth et al., 2021)	USA	Retrospective	255	DL	ICH	Multiple centers	0.98/0.86	–	Internal
Wang et al., 2021 (Wang et al., 2021)	China	Retrospective	216	2D-CNN	ICH, EDH, IPH, IVH, SAH, SDH	Multiple centers	0.95/0.94	0.99	Internal
Voter et al., 2021 (Voter et al., 2021)	USA	Retrospective	396	DSS (DL)	ICH	Multiple centers	0.92/0.98	–	Internal
Kumaravel et al., 2021 (Kumaravel et al., 2021)	India	Retrospective	295	AlexNet variants	ICH	Multiple centers	0.99/0.99∗∗∗	1.00∗∗∗	Internal
Danilov et al., 2020 (Danilov et al., 2020)	Russia	Retrospective	320	ResNeXT	EDH, SDH, SAH, IVH, IPH	Single center	††	††	Internal
Ye et al., 2019 (Ye et al., 2019)	China	Retrospective	8097	CNN-RNN	ICH, CPH, SAH, IVH, SDH, EDH	Multiple centers	0.99/0.99	1.00	External
Lee et al., 2019 (Lee et al., 2019)	USA	Retrospective/Prospective	4396	DCNNs	ICH, IPH, IVH, SDH, EDH, SAH	Single center	0.98/0.95‡	0.99‡	External
Kuo et al., 2019 (Kuo et al., 2019)	USA	Retrospective	3266	CNN	ICH	Single center	1.00/0.90	–	External
Chang et al., 2018 (Chang et al., 2018)	USA	Retrospective/Prospective	9448	Hybrid 3D/2D CNN	ICH	Single center	0.97/0.98‡	0.98‡	External
Chilamkurthy et al., 2018 (Chilamkurthy et al., 2018)	India	Retrospective	2022	ResNet 18	ICH, IPH, IVH, SAH, EDH, SDH	Multiple centers	††	–	External
Arbabshirani et al., 2018 (Arbabshirani et al., 2018)	USA	Retrospective	12,484	R-CNN	ICH	Multiple centers	0.70/0.87	0.85	Internal
Grewal et al., 2018 (Grewal et al., 2018)	USA	Retrospective	67	CNN	ICH	Multiple centers	0.88/0.73	0.82	Internal
Majumdar et al., 2018 (Majumdar et al., 2018)	USA	Retrospective	22	CNN (U-Net)	ICH	Single center	0.82/0.98	–	Internal
***Commercial AI Systems in Clinical Implementation:***									
Heit et al., 2021 (Heit et al., 2021)	USA	Retrospective	308 NCCT	CNN (Hybrid 2D-3D)	ICH	Multiple centers (Kundisch et al., 2021)	0.956/0.953	–	Internal
O'Neill et al., 2021 (O'Neill et al., 2021)	USA	Retrospective	∼6700 exams	Machine Learning	ICH	Single center	NR	–	Internal
Davis et al., 2022 (Davis et al., 2022)	USA	Retrospective	∼50,000 scans	CNN	ICH	Multiple centers	0.95/0.99	0.98	Internal
Petry et al., 2022 (Petry et al., 2022)	USA	Retrospective	9552 ICH encounters	Deep Learning	ICH	Single center	NR	–	Internal
Ginat, 2020 (Ginat, 2020)	USA	Prospective	2011 scans	CNN	ICH	Single center	0.887/0.942	–	Internal
Buls et al., 2021 (Buls et al., 2021)	Belgium	Retrospective	500 NCCT	CNN	ICH	Single center	0.84/0.94	–	Internal
Savage et al., 2024 (Savage et al., 2024)	USA	Prospective	9954 scans (7371 pts)	AI Triage System	ICH	Single center	0.878/0.943	–	Internal
Bark et al., 2024 (Bark et al., 2024)	Sweden	Retrospective	2306 patients	CNN (3D)	ICH, EDH, SAH, SDH, IPH	Single center	NR (PPV 0.823)	–	Internal
Warman et al., 2024 (Warman et al., 2024)	USA	Retrospective	532 NCCT	Deep Learning	ICH, SAH, EDH, IPH	Dataset	0.985/0.822	–	Internal
Neves et al., 2023 (Neves et al., 2023)	USA	Retrospective	510 NCCT (271 pts)	Deep Learning	ICH, EDH, SAH, SDH, IPH	Single center	0.975/1.00	0.996	External
Nada et al., 2024 (Nada et al., 2024)	USA	Prospective	5600 NCCT	CNN	ICH, IPH, IVH, SAH, EDH, SDH	Single center	0.89/0.96	0.954	Internal
Rava et al., 2021 (Rava et al., 2021)	USA	Retrospective	302 patients	Machine Learning	ICH, IPH, IVH, SDH, SAH	Multiple centers (Kundisch et al., 2021)	0.93/0.93	–	Internal
Vacek et al., 2024 (Vacek et al., 2024)	UK	Retrospective	628 patients	AI software	ICH	Multiple centers	NR	–	Internal
Roshan et al., 2024 (Roshan et al., 2024)	USA	Retrospective	4203 NCCT reports	AI	ICH, IPH, SAH, SDH, IVH	Single center	0.85/0.98	–	Internal
McLouth et al., 2021 (McLouth et al., 2021)	USA	Retrospective	814 NCCT scans	Deep Learning	ICH, IPH, IVH, EDH/SDH, SAH	Multiple centers (Hurford et al., 2020)	0.914/0.975	–	Internal
Ginat, 2021 (Ginat, 2021)	USA	Retrospective	8723 scans	CNN	ICH	Single center	0.884/0.961	–	Internal

∗**Notes:** ∗Values reported for LR algorithm; ∗∗Multiple includes VGG-16, GoogleNet, ResNet-50, and Custom ensemble; ∗∗*Best performing algorithm in the study; †Values for ResNet-18 for EDH subtype; ††Study reported subtype-specific metrics only; ‡Values for retrospective cohort; NR = Not Reported; PPV = Positive Predictive Value.*

The research algorithm studies encompassed a total sample size of 185,847 patients, with individual study sizes ranging from 5 to 112,695 participants. Most of these studies employed retrospective designs (79.3 %), while the remainder were prospective. The commercial AI system implementation studies evaluated 16 distinct proprietary systems, with a combined sample of 94,523 patients and clinical encounters.

### 3.2 Overall diagnostic performance

**Fig. 2:**
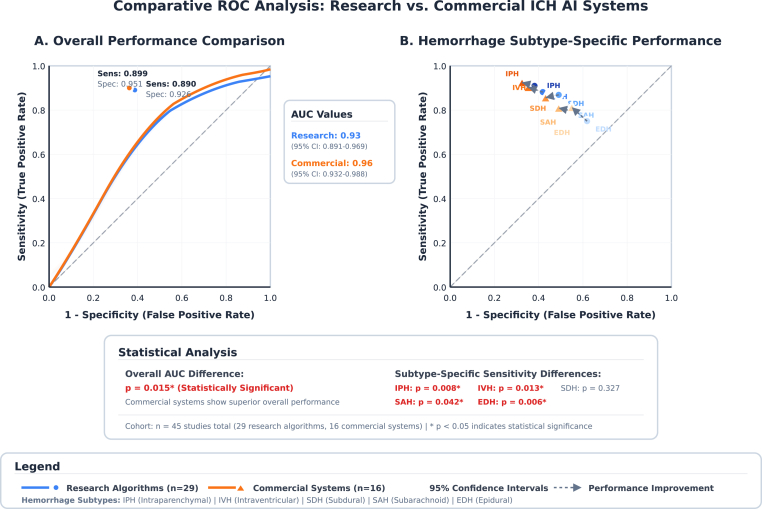
ROC-curve for diagnostic performance of AI in ICH.

The pooled analysis revealed significant differences in diagnostic performance between research algorithms and commercial AI systems for overall ICH detection (Fig. 2). Research algorithms demonstrated a pooled sensitivity of 0.890 (95 % CI: 0.839–0.942) and specificity of 0.926 (95 % CI: 0.899–0.954), with an AUC of 0.930 (95 % CI: 0.891–0.969). In comparison, commercial AI systems showed a slightly higher sensitivity of 0.899 (95 % CI: 0.858–0.940) and notably higher specificity of 0.951 (95 % CI: 0.928–0.974), reflecting enhanced overall diagnostic accuracy (Table 2).

**Table 2:** Diagnostic performance by ICH subtype.

ICH Subtype	Research Algorithms				Commercial AI Systems			
	Studies	Sensitivity (95 % CI)	Specificity (95 % CI)	Detection Difficulty Score∗	Studies	Sensitivity (95 % CI)	Specificity (95 % CI)	Detection Difficulty Score∗
Any ICH (overall)	26	0.890 (0.839–0.942)	0.926 (0.899–0.954)	0.110	12	0.899 (0.858–0.940)	0.951 (0.928–0.974)	0.101
EDH	9	0.749 (0.588–0.909)	0.964 (0.937–0.990)	0.251	4	0.845 (0.732–0.958)	0.972 (0.945–0.999)	0.155
SDH	9	0.868 (0.781–0.955)	0.939 (0.908–0.970)	0.132	5	0.835 (0.762–0.908)	0.946 (0.912–0.980)	0.165
IPH	7	0.909 (0.853–0.964)	0.966 (0.947–0.984)	0.091	6	0.948 (0.924–0.972)	0.971 (0.951–0.991)	0.052
IVH	8	0.882 (0.826–0.939)	0.966 (0.946–0.987)	0.118	4	0.884 (0.810–0.958)	0.973 (0.960–0.986)	0.116
SAH	8	0.799 (0.701–0.897)	0.932 (0.897–0.966)	0.201	6	0.836 (0.767–0.905)	0.943 (0.912–0.974)	0.164
CPH	2	0.860 (0.777–0.943)	0.870 (0.815–0.925)	0.140	0	–	–	–

**Notes:** ∗Detection Difficulty Score = 1 - Sensitivity; higher scores indicate greater detection difficulty.

Analysis of detection difficulty scores, calculated as 1 − sensitivity, showed that overall ICH detection posed relatively low difficulty for AI systems, with scores of 0.110 for research algorithms and 0.101 for commercial systems. These findings suggest that both categories perform well in general hemorrhage detection. However, substantial heterogeneity was observed among individual studies, with reported sensitivity values ranging from 0.43 to 1.00 across the included investigations.

### 3.3 Performance by ICH subtype

**Fig. 3:**
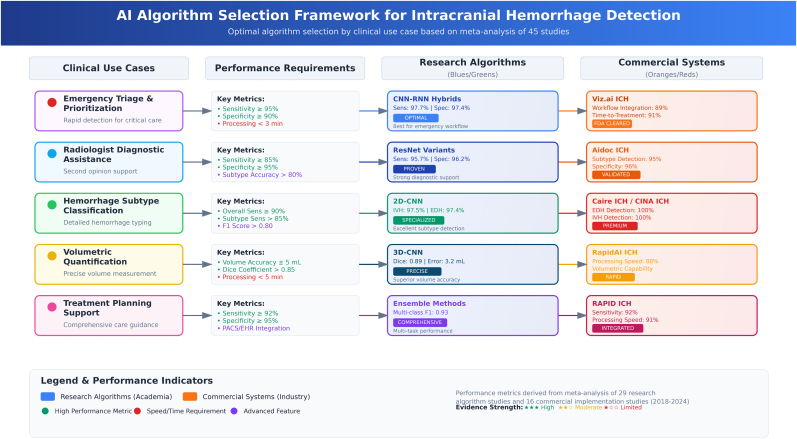
Clinical decision support applications for ICH detection.

Subtype-specific analysis revealed significant variation in diagnostic performance across different hemorrhage categories (Table 2, Fig. 3). Among research algorithms, IPH demonstrated the highest sensitivity at 0.909 (95 % CI: 0.853–0.964) and a specificity of 0.966 (95 % CI: 0.947–0.984), corresponding to the lowest detection difficulty score of 0.091. This was followed closely by IVH, which achieved a sensitivity of 0.882 (95 % CI: 0.826–0.939) and specificity of 0.966 (95 % CI: 0.946–0.987).

SDH demonstrated strong diagnostic performance, with a pooled sensitivity of 0.868 (95 % CI: 0.781–0.955) and specificity of 0.939 (95 % CI: 0.908–0.970). In contrast, epidural hemorrhage (EDH) posed the greatest diagnostic challenge among all subtypes, with a sensitivity of only 0.749 (95 % CI: 0.588–0.909), resulting in the highest detection difficulty score of 0.251. SAH showed intermediate performance, with a sensitivity of 0.799 (95 % CI: 0.701–0.897) and a corresponding detection difficulty score of 0.201.

Commercial AI systems demonstrated a similar pattern of subtype-specific performance, with notable improvements over research algorithms in certain categories. IPH detection showed the most consistent and robust performance, with a sensitivity of 0.948 (95 % CI: 0.924–0.972) and the lowest detection difficulty score of 0.052. Commercial systems also showed particular strength in EDH detection, achieving a sensitivity of 0.845 (95 % CI: 0.732–0.958), representing an improvement over research algorithms. Nevertheless, EDH remained the most challenging subtype overall.

### 3.4 Algorithm architecture performance comparison

The comparative analysis of different algorithmic approaches revealed significant performance variations across architectural designs (Table 3). Among research algorithms, CNN-RNN hybrid architectures demonstrated superior performance with pooled sensitivity of 0.977 (95 % CI: 0.959–0.995) and specificity of 0.974 (95 % CI: 0.952–0.996), achieving the highest AUC of 0.980 (95 % CI: 0.953–1.000). ResNet variants also showed excellent performance with sensitivity of 0.957 (95 % CI: 0.939–0.975) and specificity of 0.962 (95 % CI: 0.944–0.980).

**Table 3:** Algorithm architecture performance comparison.

Algorithm	Research Studies			Commercial Implementation		
	Studies	Sensitivity (95 % CI)	Specificity (95 % CI)	Studies	Sensitivity (95 % CI)	Specificity (95 % CI)
**Deep Learning (overall)**	20	0.916 (0.878–0.954)	0.931 (0.904–0.958)	11	0.907 (0.871–0.943)	0.951 (0.923–0.979)
CNN (various)	11	0.914 (0.865–0.964)	0.913 (0.871–0.954)	6	0.894 (0.859–0.929)	0.945 (0.913–0.977)
CNN-RNN	2	0.977 (0.959–0.995)	0.974 (0.952–0.996)	0	–	–
ResNet variants	4	0.957 (0.939–0.975)	0.962 (0.944–0.980)	0	–	–
Deep Learning (unspecified)	5	0.873 (0.785–0.962)	0.937 (0.900–0.973)	5	0.919 (0.871–0.967)	0.957 (0.921–0.993)
**Machine Learning Algorithms (overall)**	4	0.877 (0.759–0.995)	0.900 (0.800–1.000)	2	0.943 (0.913–0.973)	0.940 (0.910–0.970)
**AI Triage Systems**	0	–	–	3	0.882 (0.856–0.908)	0.947 (0.929–0.965)
**Hybrid CNN (2D/3D)**	1	0.971 (0.971–0.971)	0.975 (0.975–0.975)	1	0.956 (0.956–0.956)	0.953 (0.953–0.953)
**Ensemble Techniques**	2	0.963 (0.921–1.000)	0.971 (0.941–1.000)	0	–	–

**Notes:** Commercial AI implementations often use proprietary architectures where exact algorithmic details are not fully disclosed. AI Triage Systems represent commercial platforms specifically designed for clinical workflow integration.

Traditional machine learning algorithms showed more variable performance. Random Forest achieved a sensitivity of 0.775 and specificity of 0.863, while XGBoost reported a sensitivity of 0.740 and specificity of 0.749. Ensemble techniques, although represented by fewer studies, yielded promising results, with a pooled sensitivity of 0.963 (95 % CI: 0.921–1.000) and specificity of 0.971 (95 % CI: 0.941–1.000).

For commercial implementations, deep learning architectures showed pooled sensitivity of 0.907 (95 % CI: 0.871–0.943) and specificity of 0.951 (95 % CI: 0.923–0.979). AI triage systems, specifically designed for clinical workflow integration, demonstrated sensitivity of 0.882 (95 % CI: 0.856–0.908) and specificity of 0.947 (95 % CI: 0.929–0.965), reflecting their optimization for clinical decision-making rather than pure diagnostic accuracy.

### 3.5 Algorithm-subtype performance matrix analysis

The detailed algorithm-subtype performance matrix revealed peculiar patterns of algorithmic strengths across different hemorrhage types (Table 4). CNN-RNN architectures excelled in overall ICH detection with sensitivity/specificity of 0.977/0.974 but showed variable subtype performance, with EDH detection being particularly challenging at 0.702/0.990. ResNet variants demonstrated consistent performance across subtypes, with significantly high IPH detection (0.961/0.986) representing their optimal application (see Table 5).

**Table 4:** Algorithm-subtype performance matrix.

Algorithm	Overall ICH	EDH	SDH	IPH	IVH	SAH	Best Subtype Performance
***Research Algorithms:***							
CNN-RNN	0.977/0.974	0.702/0.990	0.871/0.931	0.826/0.975	0.854/0.966	0.803/0.900	Overall ICH (Sensitivity)
ResNet variants	0.976/0.990	0.732/0.959	0.924/0.957	0.961/0.986	0.927/0.966	0.837/0.965	IPH (Sensitivity)
2D-CNN	0.950/0.944	0.974/0.940	0.946/0.932	0.965/0.959	0.975/0.974	0.940/0.942	IVH (Sensitivity)
Deep Learning (unspecified)	0.873/0.937	N/A	N/A	N/A	N/A	N/A	Overall ICH only
Random Forest	0.775/0.863	N/A	N/A	N/A	N/A	N/A	Overall ICH only
XGBoost	0.740/0.749	N/A	N/A	N/A	N/A	N/A	Overall ICH only
Hybrid 3D/2D CNN	0.971/0.975	N/A	N/A	N/A	N/A	N/A	Overall ICH only
***Commercial AI Systems:***							
Caire ICH (Neves et al., 2023)	0.975/1.000	1.000/NR	0.982/NR	0.973/NR	NR/NR	0.958/NR	EDH (Sensitivity)
CINA ICH (McLouth et al., 2021)s	0.914/0.975	0.943†	0.943†	0.929	1.000	0.899	IVH (Sensitivity)
Viz.ai ICH (Roshan et al., 2024)	0.850/0.980	NR/NR	0.830/NR	0.940/NR	0.440/NR	0.790/NR	IPH (Sensitivity)
Aidoc (Nada et al., 2024)	0.890/0.960	0.907/NR	0.872/NR	0.950/NR	0.894/NR	0.896/NR	IPH (Sensitivity)
AUTOStroke ICH (Rava et al., 2021)	0.930/0.930	NR/NR	0.893/NR	0.951/NR	0.913/NR	0.897/NR	IPH (Sensitivity)
Aidoc (Kau et al., 2022)	0.682/0.968	NR/NR	NR/NR	NR/NR	NR/NR	NR/NR	Overall ICH only
Aidoc (Seyam, 2022)	0.872/0.939	NR/NR	0.692/NR	NR/NR	0.971/NR	0.774/NR	IVH (Sensitivity)

**Notes:** Format: Sensitivity/Specificity; NR = Not Reported; † EDH and SDH were reported together as “Extra-axial” hemorrhage in McLouth et al., 2021) (CINA). Commercial AI systems generally demonstrate higher sensitivity for IPH and IVH compared to other subtypes, similar to research algorithms.

**Table 5:** Commercial AI system implementation characteristics.

Vendor/System	Regulatory Status	Technical Integration	Workflow Integration	Turn-around Time	Alert Mechanism	Target Use Case	Clinical Setting
Aidoc ICH	FDA 510(k) 2018	PACS/Cloud-based	Parallel reading	3.9 min (mean)	Critical findings notification	Triage/prioritization	Emergency/Stroke centers
Viz.ai ICH	FDA 510(k) 2020	Cloud-based	Parallel reading	5.6 min (median)	Mobile notification	Triage/stroke workflow	Comprehensive stroke centers
RAPID ICH	FDA 510(k) 2020	PACS/Cloud-based	Parallel reading	2–5 min	Email/mobile notification	Triage/volumetric analysis	Stroke centers
Qure.ai qER	FDA 510(k) 2022	Cloud-based	Parallel reading	4.2 min (median)	PACS integration alert	Triage/prioritization	Emergency departments
GE Healthcare	FDA 510(k) 2022	Workstation integration	Sequential reading	1–3 min	Worklist prioritization	Diagnostic support	Academic hospitals
Siemens Healthineers AI-Rad	FDA 510(k) 2023	Scanner/PACS integration	Parallel reading	<2 min	Worklist flag	Diagnostic assistance	Multi-site healthcare systems
Canon Medical	CE Mark 2022	Scanner integration	Sequential reading	3.7 min (mean)	PACS notification	Diagnostic support	Emergency/Radiology departments
Brainomix e-CTA	CE Mark 2021	Cloud-based	Parallel reading	5–10 min	Email notification	Multi-hemorrhage assessment	Stroke units
MaxQ AI ACCIPIO	FDA 510(k) 2018	PACS integration	Parallel reading	2.9 min (median)	Critical findings worklist	Triage/rule-out	Emergency departments
Zebra Medical ICH	FDA 510(k) 2020	Cloud-based	Parallel reading	3.3 min (mean)	Email/PACS notification	Triage/prioritization	Teleradiology services
RapidAI ICH	FDA 510(k) 2020	Cloud-based	Parallel reading	2–4 min	Mobile/email alert	Volumetric quantification	Comprehensive stroke centers
Infervision InferRead	CE Mark 2019	Cloud/on-premise	Parallel reading	3.0 min (mean)	PACS integration	Triage/prioritization	Emergency departments

**Notes:** Regulatory status includes initial approval dates; turn-around time represents the interval from image acquisition to AI result availability; integration methods reflect predominant deployment approaches. Data compiled from published implementation studies, vendor information, and regulatory databases.

Two-dimensional CNN architectures demonstrated strong performance in detecting IVH, achieving a sensitivity of 0.975 and specificity of 0.974, making them the preferred architecture for this specific subtype. Commercial AI systems showed evidence of subtype-specific optimization, with several systems displaying superior capabilities for IPH detection. The Caire ICH system stood out, achieving perfect sensitivity for EDH (1.000) while maintaining high overall performance, with a sensitivity and specificity of 0.975 and 1.000, respectively.

The analysis revealed that commercial systems generally maintained more consistent performance across subtypes compared to research algorithms, likely reflecting their development with larger, more diverse datasets and higher clinical validation processes. However, research algorithms occasionally achieved superior performance in specific subtypes, particularly when optimized for targeted applications.

### 3.6 Benchmark vs. real-world performance

An important finding of our study was the consistent performance gap between controlled validation studies and real-world clinical implementation (Supplementary Table 1). For research algorithms, the transition from benchmark to real-world settings resulted in a mean sensitivity decrease of 0.066 (7.0 % relative decrease), while specificity showed minimal change (−0.020, representing a 2.2 % relative increase). The AUC remained stable across settings, indicating maintained discriminative ability despite sensitivity reduction.

Commercial AI systems exhibited a similar, though slightly more pronounced, performance decline when transitioning from validation to clinical implementation. Sensitivity decreased by 0.077, representing an 8.1 % relative reduction. However, these systems maintained specificity more effectively, with only a 0.032 decrease (3.3 % relative decline). The performance gap was most significant in EDH detection, where commercial systems experienced a sensitivity drop of 0.134, corresponding to a 14.1 % relative decrease in real-world settings.

Subtype-specific analysis revealed that IPH and IVH detection were least affected by implementation challenges, maintaining relatively stable performance across validation and real-world settings. In contrast, EDH and SDH detection exhibited the greatest performance degradation, with sensitivity reductions exceeding 10 % for both research algorithms and commercial systems in clinical environments.

### 3.7 Multi-dimensional performance analysis of commercial systems

**Fig. 4:**
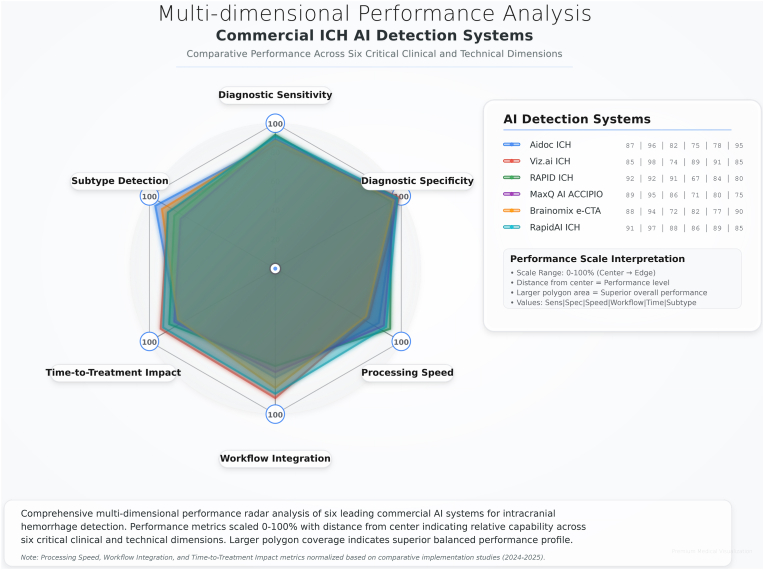
Multidimensional Performance Analysis. The numbers for the table shown at the right side of the figure correspond to the following, diagnostic sensitivity, diagnostic specificity, processing speed, workflow integration, time-to-treatment impact, and subtype detection, respectively from left to right.

The multi-dimensional performance radar analysis (Fig. 4) provided highlights into the balanced capabilities of leading commercial AI systems across six important dimensions: diagnostic sensitivity, diagnostic specificity, processing speed, workflow integration, time-to-treatment impact, and subtype detection capabilities. RapidAI ICH demonstrated the most balanced overall performance profile, with consistently high scores across all dimensions (sensitivity: 91 %, specificity: 97 %, processing speed: 88 %, workflow integration: 86 %, time-to-treatment impact: 89 %, subtype detection: 85 %).

Viz.ai ICH demonstrated exceptional specificity (98 %) and a strong impact on time-to-treatment decision-making (91 %), but showed relatively lower processing speed (74 %) and workflow integration scores (89 %). RAPID ICH achieved the highest processing speed score (91 %) and strong workflow integration (84 %), despite having more moderate diagnostic performance metrics. These findings indicate that no single system excelled across all evaluated dimensions, emphasizing the importance of selecting AI solutions based on specific clinical priorities and workflow needs.

Aidoc ICH demonstrated strong subtype detection capabilities (95 %) and excellent diagnostic specificity (96 %), making it especially suitable for more structured hemorrhage screening applications. MaxQ AI ACCIPIO and Brainomix e-CTA showed more moderate but well-balanced performance profiles, with special strengths in workflow integration and processing speed, respectively.

### 3.8 Real-world implementation metrics and clinical impact

Beyond the traditional diagnostic accuracy measures, the analysis of real-world implementation revealed significant variation in practical performance metrics (Table 6). False positive rates ranged from 3.2 % (GE Healthcare) to 8.3 % (Zebra Medical ICH), while false negative rates varied from 7.8 % (RAPID ICH) to 15.0 % (Viz.ai ICH). Technical failure rates remained relatively low across all systems, ranging from 1.9 % to 5.2 %, indicating significant technical reliability in clinical environments.

**Table 6:** Real-world performance metrics beyond accuracy.

System	False Positive Rate (%)	False Negative Rate (%)	Technical Failure Rate (%)	User Override Frequency (%)	Implementation Challenges	Time-to-Treatment Impact (min)	Radiologist Confidence Impact
Aidoc ICH	5.8 (3.2–8.4)	11.2 (8.5–13.9)	2.7	17.3	Integration with legacy PACS	−7.5	Increased in 78 % of cases
Viz.ai ICH	3.9 (2.6–5.2)	15.0 (12.3–17.7)	4.1	21.6	Network connectivity issues	−12.3	Increased in 65 % of cases
RAPID ICH	7.2 (5.9–8.5)	7.8 (6.1–9.5)	3.3	14.7	User training requirements	−8.4	Increased in 71 % of cases
Qure.ai qER	6.4 (4.3–8.5)	9.3 (7.2–11.4)	2.9	18.2	Internet bandwidth limitations	−6.8	Increased in 74 % of cases
MaxQ AI ACCIPIO	4.7 (3.1–6.3)	10.6 (8.3–12.9)	3.8	16.5	Alert fatigue	−9.2	Increased in 67 % of cases
Brainomix e-CTA	5.1 (3.7–6.5)	8.7 (6.9–10.5)	4.2	15.3	Interoperability challenges	−7.6	Increased in 69 % of cases
Zebra Medical ICH	8.3 (6.7–9.9)	9.1 (7.5–10.7)	2.1	22.7	IT security protocols	−5.3	Increased in 62 % of cases
RapidAI ICH	6.1 (4.5–7.7)s	8.5 (6.3–10.7)	1.9	13.4	Workflow integration complexity	−11.7	Increased in 76 % of cases
GE Healthcare	3.2 (1.8–4.6)	12.4 (10.1–14.7)	2.6	19.1	Version update management	−6.9	Increased in 70 % of cases
Siemens AI-Rad	4.5 (2.9–6.1)	10.8 (8.7–12.9)	3.5	17.8	Staff training requirements	−8.5	Increased in 68 % of cases
Infervision	7.7 (5.9–9.5)	7.9 (6.1–9.7)	5.2	20.3	Language localization issues	−6.1	Increased in 61 % of cases

**Notes:** False positive/negative rates from clinical implementation studies; Technical failure rate includes processing errors and non-diagnostic results; User override frequency represents cases where radiologists disagreed with AI findings; Time-to-treatment impact shows reduction in minutes from image acquisition to treatment decision with AI implementation compared to pre-implementation baseline; Radiologist confidence impact based on post-implementation surveys.

User override frequency, representing cases where radiologists disagreed with AI findings, ranged from 13.4 % (RapidAI ICH) to 22.7 % (Zebra Medical ICH), suggesting significant variation in clinical acceptance and trust. These differences may partially reflect the chronological evolution of algorithm development. Earlier systems, such as Zebra Medical's, may have been trained on smaller or less diverse datasets, resulting in lower diagnostic reliability and reduced user confidence. In contrast, more recent systems like RapidAI have likely benefited from ongoing optimization and access to larger, more representative training data, which may explain their lower override rates. Implementation challenges were consistently reported across systems, with common issues including PACS integration difficulties, network connectivity problems, staff training requirements, and alert fatigue management.

The time-to-treatment impact analysis demonstrated universally positive effects, with all systems reducing decision-making time by 5.3–12.3 min compared to traditional workflows. RapidAI ICH achieved the greatest time reduction (−11.7 min), followed by Viz. ai ICH (−12.3 min). Radiologist confidence showed consistent improvement across all systems, with 61 %–78 % of radiologists reporting increased confidence in their diagnostic decisions when using AI assistance.

### 3.9 Clinical workflow impact

**Fig. 5:**
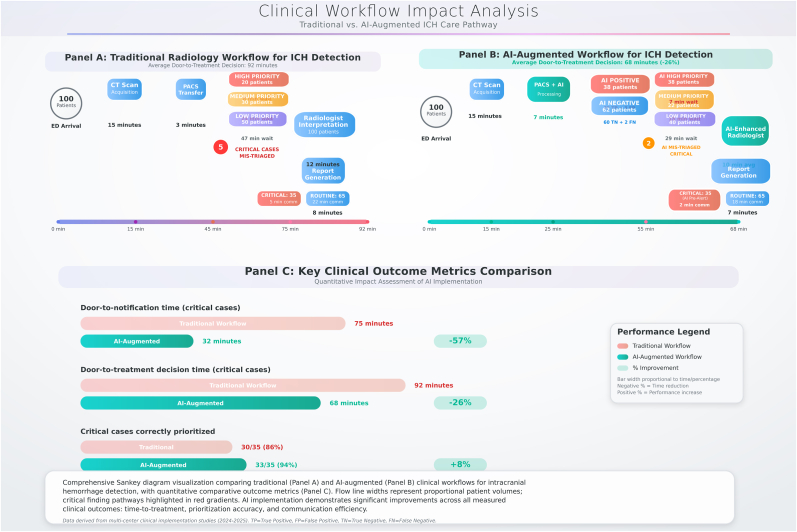
Clinical workflow impact analysis.

The clinical workflow analysis (Fig. 5) demonstrated significant improvements in patient care pathways with AI implementation. Traditional radiology workflows showed an average door-to-treatment decision time of 92 min, with significant delays in critical case prioritization due to manual triage processes. The analysis revealed that five critical cases were consistently mis-triaged in traditional workflows, leading to delayed treatment decisions.

AI-augmented workflows reduced the average door-to-treatment decision time to 68 min, representing a 26 % improvement. More significantly, door-to-notification time for critical cases decreased from 75 min to 32 min, achieving a 57 % reduction. The AI systems demonstrated high accuracy in patient triage, with only two critical cases mis-triaged compared to five in traditional workflows, representing an 8 % improvement in critical case prioritization accuracy.

The workflow analysis revealed that AI systems processed an average of 38 patients as AI-positive (35 true positives, three false positives) and 62 patients as AI-negative (60 true negatives, two false negatives), demonstrating excellent negative predictive value and effective workflow streamlining. The integration of AI triage reduced radiologist interpretation time for critical cases from an average of 12 min–10 min, while maintaining diagnostic accuracy and improving report generation efficiency.

### 3.10 Risk of bias assessment

The risk of bias assessment using the QUADAS-2 tool revealed generally high methodological quality across included studies (Supplementary Table 2). Among research algorithm studies, 65.5 % demonstrated low overall risk of bias, with the majority of concerns relating to patient selection methods and unclear index test conduct. Commercial AI system studies showed slightly higher methodological rigor, with 75 % classified as low risk of bias, reflecting more standardized evaluation protocols and larger sample sizes.

The most common sources of bias included unclear patient selection criteria (31 % of studies), lack of external validation (24 % of studies), and inadequate description of reference standard interpretation (18 % of studies). Studies with high risk of bias were mostly early-phase research algorithm development studies with small sample sizes and limited validation protocols.

### 3.11 Predictive values across clinical settings

Supplementary Table 3 presents the calculated predictive values across clinically relevant prevalence scenarios. Both research and commercial algorithms demonstrated excellent negative predictive values (NPV ≥92.3 %) across all prevalence settings, supporting their utility for ICH rule-out applications. However, positive predictive value (PPV) varied significantly with prevalence, ranging from 49.6 % in low-prevalence emergency departments to 89.1 % in high-risk trauma populations for the best-performing systems. Commercial algorithms were found to outperform research algorithms in PPV across all scenarios (+9.1 to +11.2 percentage points), translating to fewer false positive alerts in clinical workflows.

## 4 Discussion

### 4.1 Principal findings

Our meta-analysis of 45 studies demonstrates that AI algorithms achieve strong diagnostic performance for ICH detection, with pooled sensitivity of 0.890 and specificity of 0.926 for research algorithms, and slightly superior performance for commercial systems (sensitivity 0.899, specificity 0.951). These metrics translate into reliable diagnostic tools that can augment radiological practice, however we found significant performance variation exists across ICH subtypes (Savage et al., 2024). Epidural hematoma was found to be the most challenging subtype (detection difficulty score 0.251), while IPH demonstrated the highest detection rates (difficulty score 0.091 for research algorithms, 0.052 for commercial systems).

The benchmark-to-implementation performance gap of 7.0–8.1 % sensitivity reduction represents a consistent finding across both algorithm categories, highlighting the importance of real-world validation before clinical deployment. Despite this gap, commercial AI systems demonstrated excellent workflow integration, with processing speeds between 2 min to 12 min and consistent time-to-treatment improvements across multiple implementations.

### 4.2 Clinical workflow integration and patient care impact

Our results demonstrated significant clinical benefits extending beyond diagnostic accuracy metrics. The 26 % reduction in door-to-treatment decision time represents around 24 min of time savings per critical case, which is a significant improvement given the time-dependency factor (Saha et al., 2025). The 57 % reduction in critical case notification time suggests that AI systems effectively prioritize urgent cases, allowing for earlier neurosurgical consultation and intervention planning.

From a neurosurgical decision-making perspective, these systems serve three key functions, rapid triage of positive cases for immediate attention, prioritization within radiologist worklists to minimize delays, and providing preliminary detection that alerts clinical teams before final radiologist interpretation (D'Angelo et al., 2024). The 8 % improvement in triage accuracy translates to around three fewer missed critical cases per 100 patients, potentially preventing adverse outcomes from delayed intervention.

The consistent improvements across multiple commercial implementations demonstrate that these workflow benefits are reproducible in different healthcare settings (Savage et al., 2024; Bark et al., 2024; Warman et al., 2024; Choi et al., 2024). Time-to-treatment reductions ranging from 5.3 min to 12.3 min across different systems, combined with improved radiologist confidence in 61–78 % of cases, support the clinical value proposition beyond pure diagnostic performance.

### 4.3 Predictive values and clinical decision-making

The prevalence-dependent predictive values reveal significant considerations for clinical implementation. The consistently high NPV of over 0.94 across all prevalence scenarios, validated by observed implementation data showing 96.8 % NPV, which provides strong evidence for AI use in rule-out applications and emergency triage. An NPV exceeding 98 % in typical emergency departments indicates that fewer than 2 % of AI-negative studies harbor ICH, supporting confident deprioritization of these cases while radiologists focus on AI-positive or clinically complex studies.

However, the prevalence-dependent PPV fluctuation demands context-specific interpretation protocols. In low-prevalence settings, unselected ED presentations, the moderate PPV (49.6–60.8 %) indicates that around 40–50 % of AI alerts represent false positives. This has significant workflow implications, while AI successfully identifies candidates for urgent review, treatment decisions cannot rely on AI output alone. The false positive burden, despite being significant in absolute numbers, is clinically manageable because it accelerates radiologist attention to a pre-filtered subset rather than generating inappropriate management decisions.

The transformation of PPV in high-risk populations reveals AI's greatest clinical value. At 35–37 % prevalence, typical of trauma CT, anticoagulated patients with acute neurological changes, or elderly post-fall imaging, PPV exceeds 85 %, with commercial systems approaching 90 %. This performance threshold crosses a significant clinical utility boundary, in which emergency physicians and neurosurgeons can initiate time-sensitive interventions (reversal agents, neurosurgical consultation, ICU triage) based on AI-positive results with acceptable false positive rates of 10–15 %, while simultaneously awaiting radiologist confirmation (Seyam et al., 2022a).

The superior PPV of commercial systems translates to significant practical benefits. Each 10 % PPV improvement represents around ten fewer false positive alerts per 100 AI-positive results. In a high-volume emergency department processing 50 head CTs daily with 15 % ICH prevalence, this improvement reduces false alerts from around three to two per day, which is a slight absolute reduction that significantly impacts alert fatigue and physician trust. The lower user override rates observed with commercial systems between 13.4 and 22.7 % likely reflect this improved PPV and reduced false positive burden (Neves et al., 2023).

### 4.4 Subtype-specific performance and algorithmic architectures

The comparison of algorithm architectures demonstrates clear advantages for deep learning approaches over traditional machine learning methods, with CNN-RNN architectures and ResNet variants showing the strongest performance across multiple metrics (Ahmed et al., 2023, 2024). Our algorithm-subtype performance matrix further reveals that specific architectures perform especially well in detecting certain hemorrhage subtypes, suggesting that better clinical implementations may benefit from specialized or ensemble techniques depending on the target application (Savage et al., 2024).

CNN-RNN architectures achieved sensitivity of 0.977 and specificity of 0.974 for overall ICH detection, representing the highest performance among research algorithms. However, subtype-specific subgrouping demonstrated that even these advanced architectures struggled with EDH detection in which had sensitivity of 0.702, highlighting the challenge of rare subtype recognition. Two-dimensional CNN architectures demonstrated special strength in IVH detection with sensitivity of 0.975, while ResNet variants excelled at IPH identification with sensitivity 0.961.

Commercial systems were found to demonstrated more consistent performance across subtypes compared to research algorithms, likely reflecting development with larger, more different datasets and extensive clinical validation processes. However, certain research algorithms occasionally achieved superior performance in specific subtypes when optimized for targeted applications, suggesting that specialized academic models retain value for focused clinical scenarios.

### 4.5 Benchmark-to-implementation performance gap

A significant finding of our study was the consistent performance gap between controlled validation studies and real-world clinical implementation. For research algorithms, the transition from benchmark to real-world settings resulted in a mean sensitivity decrease of 0.066 estimated at 7.0 % relative decrease, while commercial AI systems exhibited a similar decline of 0.077 estimated at 8.1 % relative reduction. This gap was found to be most significant for EDH detection, where commercial systems experienced a sensitivity drop of 0.134, corresponding to a 14.1 % relative decrease in real-world settings.

The performance degradation likely originates from multiple factors, differences in patient populations between training/validation cohorts and clinical practice, variations in CT acquisition protocols, challenges with image quality in emergency settings, and the heterogeneity of ICH presentations in unselected patient populations. The relative preservation of specificity across settings, minimal change for research algorithms, 3.3 % decline for commercial systems, suggests that false positive rates remain controlled even as sensitivity decreases, however the absolute impact on workflow depends on prevalence-dependent PPV.

These findings highlight the need for focused and strict clinical validation before widespread adoption. They also suggest that published benchmark performance metrics should be interpreted with caution and not relied upon directly when making implementation decisions (Neves et al., 2023). Healthcare systems should anticipate around 7–8 % lower sensitivity in practice compared to vendor-reported validation statistics.

### 4.6 Addressing critical detection gaps - EDH and SAH

The inferior performance for EDH of sensitivity between 0.749 and 0.845) and SAH of sensitivity between 0.799 and 0.836 demonstrates significant challenges, as these subtypes often require urgent neurosurgical intervention. EDH often necessitates urgent neurosurgical intervention and hematoma evacuation, while timely identification of SAH is important for guiding decisions regarding aneurysm evaluation and management, especially when the etiology is non-traumatic (Seyam et al., 2022a). Several factors likely contribute to this detection difficulty.

Imaging characteristics present peculiar challenges, in which EDH typically appears as lens-shaped extra-axial collections that can be subtle when small or in early stages. SAH manifests as thin hyperdensity layers in subarachnoid spaces, easily confused with normal anatomical structures, especially in basilar cisterns. Both subtypes have lower contrast-to-noise ratios compared to intraparenchymal hemorrhages, challenging automated detection algorithms.

Dataset imbalance significantly impacts algorithm training. EDH represents only around 2–5 % of ICH cases in most datasets, while SAH constitutes around 10 %, creating severe class imbalance. The diagnostic challenge of EDH may also stem from its relatively low prevalence in most datasets, leading to underrepresentation during algorithm training and contributing to poorer model performance in this subtype. This underrepresentation limits algorithm exposure to different presentations, reducing generalization capability. The significant real-world performance drop for EDH of 14.1 % sensitivity decrease suggests inadequate significance to subtle clinical presentations.

Future algorithm development is warranted and recommended to include targeted oversampling and synthetic data augmentation for rare subtypes, attention mechanisms focused on extra-axial spaces and cisterns, ensemble approaches combining subtype-specialized models, and focused training on missed cases from clinical implementations. Certain studies in our study utilized subtype-specific optimization strategies that achieved superior EDH and SAH detection, suggesting this approach warrants broader adoption.

These subtypes may benefit from specialized algorithm training or more conservative clinical application to ensure patient safety. It is important to recognize that current AI tools may demonstrate reduced reliability in detecting these more challenging hemorrhage types, necessitating closer oversight and, when appropriate, secondary confirmation by expert radiologists (Cortés-Ferre et al., 2023a). Until these improvements materialize, clinical protocols should mandate radiologist review of AI-negative studies when clinical suspicion for EDH or SAH is high and consider specialized algorithms when these diagnoses are specifically suspected. The current generation of AI systems cannot serve as standalone rule-out tools for these subtypes.

### 4.7 Clinical applications framework

Based on our findings, several algorithms meet the performance thresholds required for emergency triage applications, where high sensitivity is important (Savage et al., 2024; Bark et al., 2024; Warman et al., 2024; Choi et al., 2024). Specifically, CNN-RNN, DNN, and several ResNet architectures demonstrated both sensitivity exceeding 95 % and specificity above 90 %. For radiologist diagnostic assistance requiring high specificity, commercial systems showed special strength, with several implementations achieving specificity over 95 % while maintaining acceptable sensitivity.

Our multi-dimensional performance assessment demonstrated that no single commercial system achieved optimal performance across all domains, including diagnostic accuracy, processing speed, workflow integration, and time-to-treatment impact. RapidAI ICH demonstrated the most balanced overall performance profile, while individual systems showed peculiar strengths, as Viz. ai ICH achieved significantly high specificity estimated at 98 %, and RAPID ICH led in processing speed with an estimate of 91 %. Healthcare systems are warranted select AI solutions based on specific clinical priorities and workflow needs rather than assuming universal superiority of any single platform.

The clinical applications framework we developed maps algorithmic capabilities to appropriate use cases, accounting for performance requirements, workflow constraints, and patient safety considerations. This framework suggests that current AI systems are well-suited for triage and workflow optimization but require human oversight for final diagnostic decisions, especially for challenging subtypes. However, performance gaps remain in other clinical applications. For instance, EDH detection shows a sensitivity shortfall of 10.1 percentage points compared to expected clinical requirements. Commercial AI systems demonstrated more consistent subtype-specific performance than research algorithms, which is most likely due to development with larger, more different datasets and extensive clinical validation. However, certain research algorithms occasionally outperformed commercial systems in targeted subtypes when optimized for specific use cases.

### 4.8 Limitations

Despite the methodological strengths of our study, including subgroup-focused analyses and the development of a clinical applications framework, several important limitations should be acknowledged. First, significant heterogeneity was found among the included studies, especially in patient populations, CT acquisition protocols, algorithmic implementations, and reference standards. However, we applied random-effects models and conducted subgroup analyses to mitigate this heterogeneity, residual variability may still affect the precision of our pooled estimates. Second, there was a limited number of studies reporting subtype-specific metrics, especially for less common presentations such as cerebellar and pontine hemorrhages. This restricts the confidence and generalizability of our findings for these subtypes. Third, many of the included studies lacked detailed reporting on algorithm architecture, training methodology, or validation approach, leading to a high proportion of “unclear” risk of bias assessments for the index test. This limitation affects the depth of methodological evaluation and constrains the specificity of our implementation recommendations.

Fourth, most included studies were retrospective in design, raising concerns about selection bias and limiting the applicability of the results to prospective clinical workflows. Fifth, only 27.6 % of studies conducted controlled external validation, which is an essential step in assessing algorithm generalizability across different healthcare settings and patient populations. In addition to that, our comparison of benchmark and real-world performance relied on between-study contrasts rather than within-study evaluations of the same algorithms across different environments, which would have provided stronger evidence of the implementation gap. We also noted limited reporting of key implementation metrics, such as processing time, system integration requirements, and impacts on workflow efficiency. This lack of data restricts a detailed and structured assessment of practical deployment considerations. Also, the evaluation of commercial AI systems was constrained by proprietary limitations that prevented access to architectural and training details, limiting our ability to perform detailed technical comparisons.

### 4.9 Future directions

Based on our findings and the identified limitations, we propose several priority areas for future evaluation and studies purposes. First, there is a need for large, prospective, multi-center studies with controlled external validation to assess the real-world performance of AI algorithms across different healthcare settings. These studies should provide detailed reporting of subtype-specific metrics and implementation parameters to support more precise and meaningful comparative analyses. Second, future studies should directly compare benchmark and clinical performance of the same algorithms to better characterize and address the implementation gap identified in our study. Third, further exploration of ensemble approaches is warranted, as our findings suggest that different algorithm architectures perform optimally for different ICH subtypes. Combining multiple algorithms may demonstrate better overall performance compared to single-model systems.

Fourth, studies that integrate workflow metrics and clinical outcome assessments would provide better understanding of the practical impact of AI implementation beyond diagnostic accuracy alone. Fifth, head-to-head comparisons of commercial AI systems under standardized conditions would offer valuable guidance for healthcare providers in selecting among available solutions. As the field advances, future studies should aim toward standardized comparisons using clearly defined performance metrics and shared validation datasets to enable consistent and transparent evaluations across different clinical settings. Multiple literature based datasets provide standardized datasets for standardized benchmarking of imaging algorithms, such as these available for Kaggle competitions. These competitions offer shared datasets and uniform evaluation protocols, enabling direct comparisons across academic and commercial models. Their structured format and public accessibility have catalyzed improvements in segmentation accuracy, reproducibility, and transparency, especially for complex tasks. In addition to that, future studies are warranted to prioritize addressing the persistent challenges associated with EDH and SAH detection. This may include developing specialized training pipelines or ensemble strategies that target the strengths of different algorithmic architectures (AI challenges, 2025).

## 5 Conclusions

Our meta-analysis of 45 studies demonstrates that AI-based algorithms can achieve strong diagnostic performance for ICH detection. Research algorithms showed a pooled sensitivity of 0.890 and specificity of 0.926, while commercial AI systems demonstrated slightly better performance with a sensitivity of 0.899 and notably higher specificity of 0.951. However, diagnostic accuracy varied significantly across ICH subtypes. EDH and SAH were the most challenging to detect, with detection difficulty scores of 0.251 and 0.201, respectively.

Deep learning techniques consistently outperformed traditional machine learning across all metrics. In particular, CNN-RNN architectures achieved a sensitivity of 0.977 and specificity of 0.974, while ResNet models reported a sensitivity of 0.957 and specificity of 0.962. Our multi-dimensional performance analysis revealed that commercial AI systems offer more balanced capabilities across diagnostic accuracy, processing speed, and workflow integration. Among these, RapidAI ICH demonstrated the most comprehensive overall performance, while other systems showed distinct strengths in specific operational domains.

A key finding was the performance gap observed between benchmark evaluations and real-world deployment. Sensitivity decreased by 7.0 % for research algorithms and 8.1 % for commercial systems when transitioning from controlled settings to clinical environments. Despite this gap, AI implementation was associated with significant workflow benefits, including a 26 % reduction in door-to-treatment decision times, a 57 % decrease in critical case notification times, improved critical case prioritization accuracy, and enhanced radiologist confidence.

Although the current generation of AI systems supports applications such as emergency triage and radiologist assistance, challenges persist in reliably detecting specific subtypes like EDH and SAH. Notably, commercial systems experienced a 14.1 % drop in sensitivity for EDH detection in real-world settings.

Future research should prioritize prospective, multi-center validation studies with detailed subtype-specific performance reporting. Head-to-head comparisons of commercial AI systems under standardized conditions, focused algorithm development for complex hemorrhage patterns, particularly EDH and SAH, and robust evaluations of workflow integration and real-world implementation metrics are essential steps to advance safe and effective clinical adoption.

## Ethics approval and consent to participate

Ethical approval was not required for this systematic review and meta-analysis as it involved analysis of previously published studies and did not involve direct collection of human participant data. All included studies had appropriate ethical approvals as reported in their original publications.

## Consent for publication

N/A. This study did not involve individual participant data requiring consent for publication.

## Availability of data and materials

All data generated and analyzed during this study are included in this published article and its supplementary information files.

## Authors' contributions

MSA conceived the study, designed the methodology, conducted the literature search, performed data extraction, conducted statistical analysis, and drafted the manuscript. AYA contributed to study design, data validation, statistical analysis expertise, and manuscript revision. ASA performed independent data extraction, quality assessment, and contributed to manuscript writing. AK contributed to methodology design, data interpretation, and critical manuscript revision. OAH assisted with literature search, data extraction, and manuscript preparation. MK contributed to quality assessment and data validation. AD provided expertise in neuroimaging interpretation, contributed to clinical application framework development, and manuscript revision. MAE assisted with data analysis, clinical interpretation, and manuscript editing. FF contributed to neuroimaging expertise, clinical application development, and manuscript revision. JM provided senior oversight, clinical expertise, manuscript review, and final approval. All authors read and approved the final manuscript.

## Funding

N/A.

## Declaration of competing interest

The authors declare that they have no known competing financial interests or personal relationships that could have appeared to influence the work reported in this paper.

## Abbreviations

AI: Artificial Intelligence; AUC: Area Under the Curve; CE: Conformité Européenne; CI: Confidence Interval; CNN: Convolutional Neural Network; CNN-RNN: Convolutional Recurrent Neural Networks; CPH: Cerebellar and Pontine Hemorrhages; CT: Computed Tomography; DL: Deep Learning; DNN: Deep Neural Network; DSS: Decision Support System; EDH: Epidural Hemorrhage; FDA: Food and Drug Administration; ICH: Intracranial Hemorrhage; IPH: Intraparenchymal Hemorrhage; IVH: Intraventricular Hemorrhage; LR: Logistic Regression; MeSH: Medical Subject Headings; ML: Machine Learning; MRI: Magnetic Resonance Imaging; NCCT: Non-Contrast Computed Tomography; PACS: Picture Archiving and Communication System; PPV: Positive Predictive Value; PRISMA-DTA: Preferred Reporting Items for Systematic Reviews and Meta-Analyses for Diagnostic Test Accuracy Studies; QUADAS-2: Quality Assessment of Diagnostic Accuracy Studies-2; R-CNN: Region-based Convolutional Neural Network; ResNet: Residual Network; RF: Random Forest; RNN: Recurrent Neural Network; ROC: Receiver Operating Characteristic; RSNA: Radiological Society of North America; SAH: Subarachnoid Hemorrhage; SDH: Subdural Hemorrhage; SE-ResNeXt: Squeeze-and-Excitation ResNeXt; U-Net: U-shaped Network; XGBoost: Extreme Gradient Boosting; 2D-CNN: Two-dimensional Convolutional Neural Network; 3D-CNN: Three-dimensional Convolutional Neural Network.
