# Supplementary material for: Diagnostic performance and clinical applications of artificial intelligence for intracranial bleeding detection: A meta-analysis
Source: Brain Spine. 2025 Nov 10;5:105866. doi: 10.1016/j.bas.2025.105866 (PMC12657341; doi:10.1016/j.bas.2025.105866)
Supplement: Multimedia component 1 [file mmc1.docx]

**Supplementary Table 1:** Benchmark vs. Real-World Performance Gaps.

| **Parameter** | **Research Algorithms** | | | | **Commercial AI Systems** | | | |
| --- | --- | --- | --- | --- | --- | --- | --- | --- |
|  | **Benchmark Setting (n=8)** | **Real-world Setting (n=21)** | **Performance Gap** | **Relative Decrease (%)** | **Validation Studies (n=5)** | **Clinical Implementation (n=11)** | **Performance Gap** | **Relative Decrease (%)** |
| ***Overall ICH Detection*** | | | | | | | | |
| Sensitivity | 0.945 (0.914-0.976) | 0.879 (0.826-0.932) | 0.066 | 7.0% | 0.956 (0.931-0.981) | 0.879 (0.837-0.921) | 0.077 | 8.1% |
| Specificity | 0.902 (0.837-0.966) | 0.922 (0.889-0.955) | -0.020 | -2.2% | 0.975 (0.954-0.996) | 0.943 (0.915-0.971) | 0.032 | 3.3% |
| AUC | 0.928 (0.840-1.000) | 0.928 (0.880-0.975) | 0.000 | 0.0% | 0.979 (0.957-1.000) | 0.954 (0.923-0.985) | 0.025 | 2.6% |
| ***Performance by ICH Subtype:*** | | | | | | | | |
| EDH Sensitivity/Specificity | 0.715/0.966 | 0.757/0.993 | -0.042/-0.027 | -5.9%/-2.8% | 0.952/0.987 | 0.818/0.966 | 0.134/0.021 | 14.1%/2.1% |
| SDH Sensitivity/Specificity | 0.916/0.964 | 0.815/0.953 | 0.101/0.011 | 11.0%/1.1% | 0.941/0.967 | 0.794/0.938 | 0.147/0.029 | 15.6%/3.0% |
| IPH Sensitivity/Specificity | 0.955/0.971 | 0.858/0.982 | 0.097/-0.011 | 10.2%/-1.1% | 0.964/0.980 | 0.929/0.963 | 0.035/0.017 | 3.6%/1.7% |
| IVH Sensitivity/Specificity | 0.954/0.982 | 0.858/0.978 | 0.096/0.004 | 10.1%/0.4% | 0.936/0.990 | 0.847/0.964 | 0.089/0.026 | 9.5%/2.6% |
| SAH Sensitivity/Specificity | 0.855/0.964 | 0.758/0.932 | 0.097/0.032 | 11.3%/3.3% | 0.897/0.972 | 0.810/0.929 | 0.087/0.043 | 9.7%/4.4% |

***Notes:*** *Research algorithm data from original meta-analysis. Commercial AI data extracted from 16 studies with reported performance metrics. "Validation Studies" refers to vendor-controlled or initial validation studies, while "Clinical Implementation" refers to independent clinical evaluations in real-world settings. The performance gap for commercial systems follows a similar pattern to research algorithms, with slightly larger implementation gaps observed for EDH and SDH.s*
