# Supplementary material for: Diagnostic performance and clinical applications of artificial intelligence for intracranial bleeding detection: A meta-analysis
Source: Brain Spine. 2025 Nov 10;5:105866. doi: 10.1016/j.bas.2025.105866 (PMC12657341; doi:10.1016/j.bas.2025.105866)
Supplement: Multimedia component 2 [file mmc2.docx]

**Supplementary Table 2:** Risk of Bias Assessment for Included Studies Using QUADAS-2 Tool.

| **Author, Year** | **Patient Selection** | **Index Test** | **Reference Standard** | **Flow and Timing** | **Overall Risk** |
| --- | --- | --- | --- | --- | --- |
| ***Research Algorithm Development and Validation Studies:*** | | | | | |
| Schmitt et al., 2022 | Unclear | Low | Low | Low | Low |
| Phaphuangwittayakul et al., 2022 | Low | Low | Low | Low | Low |
| Hopkins et al., 2022 | Low | Unclear | Low | Low | Low |
| Seyam et al., 2022 | Low | Low | Low | Low | Low |
| Altuve & Pérez, 2022 | Unclear | Low | Low | Unclear | Unclear |
| Tang et al., 2022 | Low | Low | Low | Low | Low |
| Cortes-Ferre et al., 2022 | Low | Low | Low | Low | Low |
| Kau et al., 2022 | Low | Low | Low | Low | Low |
| Tharek et al., 2022 | Low | Low | Low | Low | Low |
| Abe et al., 2022 | High | Low | Low | Low | High |
| Trevisi et al., 2022 | Unclear | Low | Low | Low | Unclear |
| Uchida et al., 2022 | Low | Unclear | Low | Low | Unclear |
| Alis et al., 2022 | Low | Low | Low | Low | Low |
| Rao et al., 2022 | Unclear | Low | Low | Low | Unclear |
| Zhou et al., 2022 | Low | Low | Low | Low | Low |
| Salehinejad et al., 2021 | Low | Low | Low | Unclear | Unclear |
| McLouth et al., 2021 | Low | Unclear | Low | Low | Unclear |
| Wang et al., 2021 | Unclear | Low | Low | Low | Unclear |
| Voter et al., 2021 | Low | Low | Low | Low | Low |
| Kumaravel et al., 2021 | Unclear | Low | Low | Low | Unclear |
| Danilov et al., 2020 | Unclear | Low | Low | Low | Unclear |
| Ye et al., 2019 | Low | Low | Low | Low | Low |
| Lee et al., 2019 | Low | Low | Low | Low | Low |
| Kuo et al., 2019 | Low | Low | Low | Low | Low |
| Chang et al., 2018 | Low | Low | Low | Low | Low |
| Chilamkurthy et al., 2018 | Low | Low | Low | Low | Low |
| Arbabshirani et al., 2018 | Unclear | Low | Low | Low | Unclear |
| Grewal et al., 2018 | Unclear | Low | Low | Unclear | Unclear |
| Majumdar et al., 2018 | High | Low | Low | Low | High |
| ***Commercial AI Systems in Clinical Implementation:*** | | | | | |
| Savage et al., 2024 | Low | Low | Low | Low | Low |
| Bark et al., 2024 | Unclear | Unclear | Low | Unclear | Unclear |
| Warman et al., 2024 | Low | Low | Low | Low | Low |
| Nada et al., 2024 | Low | Low | Low | Low | Low |
| Vacek et al., 2024 | Low | High | Low | Low | High |
| Roshan et al., 2024 | Low | Low | Low | Low | Low |
| Neves et al., 2023 | Low | Low | Low | Low | Low |
| Davis et al., 2022 | Unclear | Low | High | Low | High |
| Petry et al., 2022 | High | Unclear | High | Low | High |
| Heit et al., 2021 | Low | Low | Low | Low | Low |
| O'Neill et al., 2021 | Low | Unclear | Low | Low | Unclear |
| Buls et al., 2021 | Low | Low | Low | Low | Low |
| Rava et al., 2021 | Low | Low | Low | Low | Low |
| McLouth et al., 2021 (CINA) | Low | Low | Low | Low | Low |
| Ginat, 2021 | Low | Low | Low | Low | Low |
| Ginat, 2020 | Low | Low | Low | Low | Low |

***Notes:*** *Risk of bias assessment based on QUADAS-2 tool. Patient Selection: risk of bias due to patient selection methods and inclusion/exclusion criteria. Index Test: risk of bias in the conduct or interpretation of the AI algorithm. Reference Standard: risk of bias in the reference standard and its interpretation. Flow and Timing: risk of bias due to patient flow or timing of tests. Overall Risk: highest risk category assigned to the study.*
