# Supplementary material for: Diagnostic performance and clinical applications of artificial intelligence for intracranial bleeding detection: A meta-analysis
Source: Brain Spine. 2025 Nov 10;5:105866. doi: 10.1016/j.bas.2025.105866 (PMC12657341; doi:10.1016/j.bas.2025.105866)
Supplement: Multimedia component 3 [file mmc3.docx]

**Supplementary Table 3:** Predictive Values of AI Algorithms Across Clinical Prevalence Scenarios

| **Clinical Setting** | **System Type** | **Prevalence** | **Sensitivity** | **Specificity** | **PPV (95% CI)** | **NPV (95% CI)** | **False Positive Rate** | **False Negative Rate** |
| --- | --- | --- | --- | --- | --- | --- | --- | --- |
| Low-prevalence ED | Commercial | 8% | 0.899 | 0.951 | 60.8% (54.2-67.1) | 99.0% (98.5-99.3) | 39.2% | 1.0% |
|  | Research | 8% | 0.890 | 0.926 | 49.6% (43.4-55.8) | 98.9% (98.4-99.2) | 50.4% | 1.1% |
| Typical ED | Commercial | 15% | 0.899 | 0.951 | 75.5% (70.6-79.8) | 97.9% (97.2-98.5) | 24.5% | 2.1% |
|  | Research | 15% | 0.890 | 0.926 | 66.2% (61.0-71.0) | 97.7% (96.9-98.3) | 33.8% | 2.3% |
| High-risk/Trauma | Commercial | 35% | 0.899 | 0.951 | 89.1% (86.3-91.4) | 93.7% (92.1-95.0) | 10.9% | 6.3% |
|  | Research | 35% | 0.890 | 0.926 | 85.1% (81.9-87.8) | 92.7% (90.9-94.2) | 14.9% | 7.3% |
| Implementation scenario | Observed | 37% | 0.946 | 0.952 | 92.1% (79.2-97.3) | 96.8% (88.8-99.4) | 7.9% | 3.2% |
|  | Commercial | 37% | 0.899 | 0.951 | 89.7% (86.9-92.0) | 93.3% (91.6-94.7) | 10.3% | 6.7% |
|  | Research | 37% | 0.890 | 0.926 | 86.0% (83.0-88.6) | 92.3% (90.4-93.8) | 14.0% | 7.7% |

***Abbreviations:*** *CI, confidence interval; ED, emergency department; NPV, negative predictive value; PPV, positive predictive value.*
